# S08-3 Physical activity, sedentary behavior and sleep-time of Eupasmos-project participants in 16 European countries: European Union Physical Activity and Sport Monitoring System (EUPASMOS) project

**DOI:** 10.1093/eurpub/ckac093.042

**Published:** 2022-08-29

**Authors:** Henri Vähä-Ypyä

**Affiliations:** UKK Institute for Health Promotion Research, Tampete, Finland

**Keywords:** sedentary behavior, accelerometer, sleep, Europe

## Abstract

**Background:**

Increasing number of studies use device-based measurements to report levels of physical activity (PA) and sedentary behavior (SB) of populations. The studies have used different devices, device placements and analysis methods, and thus the comparison of the results between studies and populations is very challenging. The purpose of the present study was to present preliminary results of PA, SB and sleep-time measured with a similar method among adults of 16 European countries.

**Methods:**

The study is based on the Eupasmos-project where PA, SB and sleep-time of the participants (n = 6674) were measured by a tri-axial accelerometer 24/7 (UKK RM42, UKK Terveyspalvelut Oy, Tampere, Finland). Accelerometer was worn on an elastic hip-band during waking-hours and on a wrist-band attached to a non-dominant wrist during bed-time (sleeping). PA-parameters were based on mean amplitude deviation (MAD) of acceleration analyzed in 1min exponential moving average (epoch length 6s). Assessment of SB (e.g. sitting and laying down) and standing were based on the angle for posture estimation (APE). Sleep measurement was based on the movement of a non-dominant wrist during bed-time. Parameters analyzed were total daily times and different bout lengths of SB, standing, light PA, moderate-to-vigorous PA, sleep and mean number of daily steps.

**Results:**

The preliminary analysis included 47-1952 participants/country. In all countries participants spent most of their waking-hours sedentary. On weekdays the country-mean of SB varied from 8h18min to 10h7min. Participants took on average 6907-9716 steps/day. Light PA varied from 3h46min to 5h10min and moderate-to-vigorous PA from 45 to 78min. Participants wore the device on their wrist (sleeping-time) 6h42min-8h37min on weekdays. Although the total times of these behaviors were quite similar between the countries, the variation increased when different bout lengths were analyzed: the longer the bout length, the larger the variation. The hour-by-hour pattern of these behaviors varied a lot between the countries as well.

**Conclusions:**

General pattern of SB, PA and sleep-time was quite similar in all countries, but the deeper analysis revealed clear differences. To be able to compare levels and patterns of these behaviors it is essential to use the exactly same method in all participating countries.

